# A Web-Based Intervention to Help Caregivers of Older Adults With Dementia and Multiple Chronic Conditions: Qualitative Study

**DOI:** 10.2196/aging.8475

**Published:** 2018-04-23

**Authors:** Jenny Ploeg, Carrie McAiney, Wendy Duggleby, Tracey Chambers, Annie Lam, Shelley Peacock, Kathryn Fisher, Dorothy Anne Forbes, Sunita Ghosh, Maureen Markle-Reid, Jean Triscott, Allison Williams

**Affiliations:** ^1^ School of Nursing Faculty of Health Sciences McMaster University Hamilton, ON Canada; ^2^ Aging, Community & Health Research Unit McMaster University Hamilton, ON Canada; ^3^ Department of Health, Aging & Society McMaster University Hamilton, ON Canada; ^4^ Department of Psychiatry and Behavioural Neurosciences Faculty of Health Sciences McMaster University Hamilton, ON Canada; ^5^ Program for Interprofessional Practice, Education and Research Faculty of Health Sciences McMaster University Hamilton, ON Canada; ^6^ Faculty of Nursing University of Alberta Edmonton, AB Canada; ^7^ Innovations in Seniors' Care Research Unit Faculty of Nursing University of Alberta Edmonton, AB Canada; ^8^ College of Nursing University of Saskatchewan Saskatoon, SK Canada; ^9^ Faculty of Health Sciences Western University London, ON Canada; ^10^ Department of Medical Oncology Faculty of Medicine & Dentistry University of Alberta Edmonton, AB Canada; ^11^ Department of Mathematical & Statistical Sciences University of Alberta Edmonton, AB Canada; ^12^ CancerControl Alberta Health Services Edmonton, AB Canada; ^13^ Department of Health Research Methods, Evidence & Impact McMaster University Hamilton, ON Canada; ^14^ Department of Family Medicine Faculty of Medicine & Dentristry University of Alberta Edmonton, AB Canada; ^15^ Division of Care of the Elderly Faculty of Medicine & Dentistry University of Alberta Edmonton, AB Canada; ^16^ School of Geography & Earth Sciences Faculty of Science McMaster University Hamilton, ON Canada

**Keywords:** Internet, Web-based interventions, qualitative research, caregivers, aged, dementia, multiple chronic conditions

## Abstract

**Background:**

Caregivers (ie, family members and friends) play a vital role in the ongoing care and well-being of community-living older persons with Alzheimer disease and related dementia in combination with multiple chronic conditions. However, they often do so to the detriment of their own physical, mental, and emotional health. Caregivers often experience multiple challenges in their caregiving roles and responsibilities. Recent evidence suggests that Web-based interventions have the potential to support caregivers by decreasing caregiver stress and burden. However, we know little about how Web-based supports help caregivers.

**Objective:**

The objectives of this paper were to describe (1) how the use of a self-administered, psychosocial, supportive, Web-based Transition Toolkit, My Tools 4 Care (MT4C), designed by atmist, Edmonton, Alberta, Canada, helped caregivers of older adults with Alzheimer disease and related dementia and multiple chronic conditions; (2) which features of MT4C caregivers found most and least beneficial; and (3) what changes would they would recommend making to MT4C.

**Methods:**

This study was part of a larger multisite mixed-methods pragmatic randomized controlled trial. The qualitative portion of the study and the focus of this paper used a qualitative descriptive design. Data collectors conducted semistructured, open-ended, telephone interviews with study participants who were randomly allocated to use MT4C for 3 months. All interviews were audio-taped and ranged from 20 to 40 min. Interviews were conducted at 1 and 3 months following a baseline interview. Qualitative content analysis was used to analyze collected data.

**Results:**

Fifty-six caregivers from Alberta and Ontario, Canada, participated in either one or both of the follow-up interviews (89 interviews in total). Caregivers explained that using MT4C (1) encouraged reflection; (2) encouraged sharing of caregiving experiences; (3) provided a source of information and education; (4) provided affirmation; and for some participants (5) did not help. Caregivers also described features of MT4C that they found most and least beneficial and changes they would recommend making to MT4C.

**Conclusions:**

Study results indicate that a self-administered psychosocial supportive Web-based resource helps caregivers of community-dwelling older adults with Alzheimer disease and related dementia and multiple chronic conditions with their complex caregiving roles and responsibilities. The use of MT4C also helped caregivers in identifying supports for caring, caring for self, and planning for future caregiving roles and responsibilities. Caregivers shared important recommendations for future development of Web-based supports.

## Introduction

### Background

For the majority of older persons living at home with Alzheimer disease and related dementias (ADRD), family and friends take on the role of caregivers [1,2]. The trajectory of caring for a person with ADRD is typically a long process that evolves over time and intensifies, becoming more time-consuming as well as physically and emotionally demanding as the disease progresses [1]. This care is made even more complex by the presence of multiple chronic conditions (MCC), defined as 2 or more concurrent chronic conditions. A recent retrospective cohort study of community-living older adults in Ontario, Canada, found that 83% of people with dementia had 2 or more chronic conditions [3], which is even higher than people with diabetes, 76% of whom have 2 or more chronic conditions [4]. In a recent study that involved caregivers of community-living older adults with 3 or more chronic conditions, with at least one of dementia, diabetes, or stroke, the experience of managing MCC was described by caregivers as a complex, overwhelming, draining, and complicated process [5]. While caring for a person with ADRD can provide a source of meaning and fulfillment [1], its adverse effects on the caregiver’s physical and mental health, financial well-being, and quality of life are well documented [6-9]. Effective, innovative and cost-efficient interventions are needed to address the deleterious effects of caregiving and to support caregivers in caring for persons with ADRD and MCC. Web-based interventions offer a potentially low-cost and accessible way to help these caregivers.

### Use of Web-Based Resources Among Caregivers

There is growing evidence that Web-based interventions are cost-effective, efficient, and offer the potential of greater accessibility to caregivers [10]. Caregivers of persons with dementia may favor Web-based tools over face-to-face meetings because of lack of time; concerns with privacy; the need to travel, leave, or arrange care; and stigma [11]. A number of recent systematic reviews have examined the characteristics and effectiveness of Web-based interventions for caregivers of community-dwelling people living with dementia [12-16]. The components and delivery of the interventions are heterogeneous, ranging from websites with information and support to a combination of information with email, support by a coach, or exchange with other caregivers online [12]. Web-based interventions have positive effects on the well-being of caregivers, such as reductions in stress, burden, and depression [12,14]. Caregivers who used Web-based interventions reported increased confidence and self-efficacy in caregiving [12,14]. A recent systematic review examined social support interventions, including Web-based supports, for caregivers of persons with dementia [13]. Qualitative findings revealed that Web-based social interventions allowed for sharing and companionship and reduced social isolation and improved relationship quality with the person with ADRD [13].

Some studies have reported qualitative findings on caregivers’ experiences in using Web-based tools and have revealed that caregivers valued the convenience and flexibility of these tools [17-21]. Many caregivers gained knowledge and skills and learned strategies that helped them care for the person with dementia [17,19,20,22]. Some studies cited the opportunity to interact and share with, and learn from other caregivers (ie, via email, online discussion groups, blogs, or video meetings) as a benefit to the Web-based intervention [20,22]. Caregivers appreciated (1) being able to discuss their situation and express themselves freely [18,21,23]; (2) hearing the stories of other caregivers [22,23], and (3) receiving support from other caregivers [20]. Another frequently cited benefit of Web-based interventions was the opportunity to access caregiving support and advice from professionals [18,21-23].

Conversely, caregivers identified aspects of Web-based interventions that were not beneficial. These included (1) a lack of, or limited interaction with, other caregivers [18-20]; (2) difficulty with language and computer literacy [23]; and (3) difficulty with navigation [19] or specific aspects of the website.

**Figure 1:**
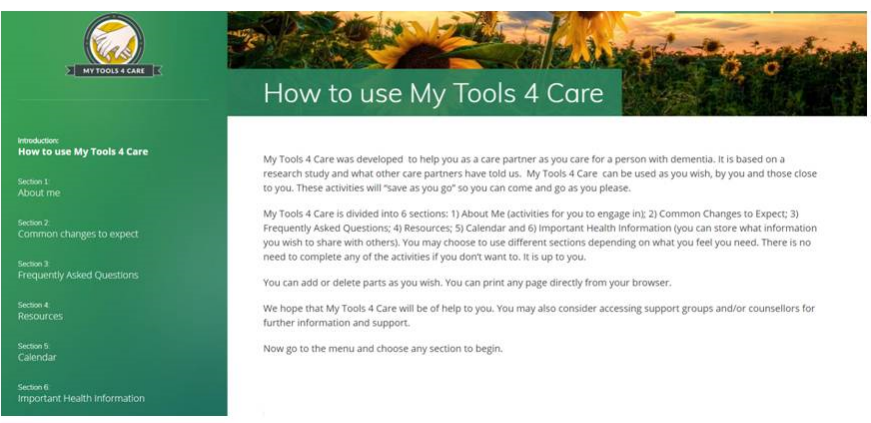
Screenshot of MyTools4Care.

We know little from a qualitative perspective of caregivers’ experiences with using Web-based interventions and how the use of these supports helps caregivers. This study is important in providing in-depth qualitative insight into how a psychosocial, Web-based intervention may help caregivers in caring for persons living with ADRD and MCC. Recommendations made by caregivers will be valuable in shaping the development of future Web-based interventions.

### Description of Intervention: My Tools 4 Care

This study is part of a larger multisite, pragmatic, mixed methods randomized controlled trial (RCT) [24]. The purpose of the RCT was to determine how the use of a self-administered, psychosocial, supportive, Web-based Transition Toolkit, My Tools 4 Care (MT4C), designed by atmist, Edmonton, Alberta, Canada, affects health-related quality of life, hope, and self-efficacy among caregivers of older adults (≥65 years) with ADRD and MCC compared with an educational control group. The Transition Toolkit developed by Duggleby, Swindle, and Peacock [25], was based on transitions theory [26].

The original paper-based Transition Toolkit was assessed in a pilot study and found to be feasible, easy to use, acceptable for use, and to have the potential to support caregivers through transitions [25]. On the basis of the pilot study, it was converted into a Web-based format: MT4C. MT4C consists of 6 sections: About Me, Common Changes to Expect, Frequently Asked Questions, Resources, Important Health Information, and a Calendar (see Figure 1). MT4C is self-administered and designed to be used by the caregiver when and how they wish. In the About Me section, users have the option to add content, such as their personal thoughts or reflections about their story and goals as a caregiver, and to store or upload information about themselves or the care recipient. The site offers information about changes that caregivers may experience, answers to frequently asked questions, weblinks to information and resources, and links to videos and written comments that capture the experiences of other caregivers. A detailed description of the sections and content of the Transition Toolkit is available in the protocol paper [24].

### Research Questions

This paper reports on the qualitative findings from participants who were randomly assigned to use MT4C for 3 months as part of the larger RCT. The research questions for the qualitative portion of the study were as follows:

1. How does a self-administered psychosocial, Web-based Transition Toolkit (MT4C) help caregivers of community-dwelling older adults with ADRD and MCC?
2. Which features of the Toolkit do caregivers find most and least helpful?
3. What changes would caregivers recommend making to the Toolkit?

## Methods

### Study Design

A qualitative descriptive design was used [27,28]. Qualitative description is based on the theoretical foundation of naturalistic inquiry, which aims to study events and persons in their natural state. The methodology aims to provide an accurate description of the phenomenon using everyday language.

### Sample

Consistent with the qualitative descriptive approach, a combination of criterion and maximum variation sampling techniques were used to obtain a purposeful sample (N=56) of caregivers of older adults with ADRD and MCC who were living at home, across both study sites (Alberta and Ontario, Canada). Unpaid caregivers who met the following criteria were eligible to participate in the larger RCT and included (1) a family member or friend who was providing physical, emotional, or financial care to an older adult (≥65 years of age) who had ADRD and 2 or more chronic conditions and was living at home; (2) English-speaking; (3) ≥18 years of age; and (4) able to use and had access to a computer with internet connection and had an email address. We used maximum variation sampling to achieve a broad representation of caregivers by gender, relationship with the care recipient (eg, spouse, child, other), and province.

### Recruitment

For the RCT, eligible participants were primarily recruited through local branches of the Alzheimer Society in both provinces. Site-specific research staff attended caregiver education groups or events held by the Alzheimer Society, to share information about the study and recruit potential participants. Coordinators from other community-based caregiver support groups, geriatric outpatient or memory clinics, adult day programs, and senior support services assisted in distributing recruitment materials (eg, brochures or postcards) to interested caregivers and referred them to the appropriate research staff in each site. With their consent, interested caregivers were contacted by the research coordinator to answer their questions, confirm their eligibility to participate in the study, and arrange a time for the first interview.

### Data Collection

From June 2015 to October 2016, data collectors conducted semistructured, open-ended telephone interviews with study participants. All interviews were audio-taped and ranged from 20 to 40 min. Interviews were conducted at 1 and 3 months following the baseline interview. The 1-month interview captured participants’ early response to MT4C, while the 3-month interview captured their response just as their access to MT4C ended. Building on the earlier work of Duggleby and colleagues [25,29], a qualitative interview guide was developed to ask participants to describe (1) how the use of MT4C helped them; (2) the features of MT4C that they found most and least helpful; and (3) what changes they would recommend making to the Toolkit (see Textbox 1).

There were 3 data collectors in Ontario and 2 in Alberta. Data collectors were health care providers and trainees and received training in conducting qualitative interviews from the project leads before they began data collection. Project leads reviewed selected interview transcripts throughout the data collection period to ensure that questions and probes were used appropriately and that data collectors were able to engage caregivers in discussion and obtain rich information. The average duration of interviews was similar across data collectors.

### Data Analysis

All interviews were transcribed verbatim by an experienced transcriptionist and then cleaned by a research assistant. Analysis was completed by a subgroup (n=4) of the larger research team. Consistent with a qualitative descriptive design, qualitative content analysis was used to analyze the data [27,28]. We used a conventional content analysis approach where coding categories were derived directly from the text data [30]. The research questions provided a broad frame for the categorization of the data. Initially, all 4 members of the analysis team independently read each transcript, looking for similarities, differences and patterns in the data, and labeling them with codes [31]. Members then met regularly to discuss, compare, corroborate, and revise codes and group them into themes describing the caregiver’s experience. NVivo 11 software (QSR International Inc., Burlington, MA) was used to manage and support analysis of the study data.

Several strategies were used to enhance the rigor of the study and ultimately produce an accurate description of the experience of using MT4C among caregivers of older adults with ADRD and MCC. Credibility, or the accuracy of the description of the caregivers’ experiences, was enhanced by purposefully sampling caregivers of community-living older adults with ADRD and MCC.

Conducting semistructured individual telephone interviews with caregivers at 1 and 3 months helped to establish trust between the interviewer and the interviewee and provided participants the opportunity to discuss their experiences from their own perspectives. Data collectors’ written field notes from each interview helped them to recall participants’ characteristics, understand the context of the interview, and follow up on the information that caregivers provided. The transcription of each recorded interview ensured that participants’ words and perspectives were represented accurately. Meeting frequently as a team to review data coding and analysis as well as maintaining a coding journal ensured that codes remained data driven and that decisions related to analysis were tracked.

### Ethics

The study was approved by the Hamilton Integrated Research Ethics Board in Hamilton, Ontario (#15-309) and by the Health Research Ethics Board—Health Panel at the University of Alberta (Pro000048721) in Edmonton, Alberta. Trained, site-specific data collectors obtained participants’ informed verbal consent before each telephone interview. A copy of the study information and consent form was sent by email to each participant immediately following the baseline interview.

**Interview guide for qualitative interviews with caregivers allocated to use My Tools 4 Care (MT4C) at 1 and 3 months post baseline.**

1. Describe any significant changes you experienced as a caregiver in the past 3 months.
2. What were you thinking about when you worked on My Tools 4 Care?
3. Did it help you deal with significant changes you experienced as a caregiver? Why or why not?
4. Did anything influence your ability to work on My Tools 4 Care?
5. Who do you think would benefit most from My Tools 4 Care?
6. What did you like best? What did you like least?
7. Do you have any other suggestions or anything else to add?

## Results

### Caregivers’ Demographic Characteristics

Fifty-six caregivers completed at least 1 semistructured telephone interview; 44 one-month interviews and 45 three-month interviews were completed. Caregivers ranged in age from 22 to 91 years, with a mean age of 64 years (SD 13) at the baseline interview. Most caregivers were female (77%, 43/56), married (84%, 47/56), and reported having medical conditions (79%, 44/56; see Table 1). While over half of these caregivers (55%, 31/56) were the spouse of the person with ADRD and MCC, a large proportion of participants were children of the care recipient (39%, 22/56). Most caregivers (77%, 43/56) lived with the person with ADRD and MCC for whom they provided care, and most (70%, 39/56) reported receiving some assistance with caregiving that is, informal (family) or formal (home care) support. Half of the study participants had been providing care for 3 or more years (50%, 28/56), whereas the other half had been in their caregiving role for 2 years or less (50%, 28/56).

Care recipients ranged from 65 to 95 years of age, with a mean age of 80 years (SD 8). Care recipients had between 2 and 17 (mean=9, SD 4) chronic conditions, in addition to ADRD. The most commonly reported chronic conditions were bowel or bladder incontinence (63%, 35/56); hypertension (59%, 33/56); arthritis, and osteoarthritis or osteoporosis (57%, 32/56).

### Qualitative Findings

Caregivers explained that using MT4C (1) encouraged reflection; (2) encouraged sharing of caregiving experiences; (3) provided a source of information and education; (4) provided affirmation; and for some participants (5) did not help. Illustrative quotes have been labeled with the participant number and the time of the interview (ie, M1=Month 1 or M3=Month 3, accordingly).

#### Using My Tools 4 Care Encouraged Reflection

Participants who used MT4C noted that many of the activities in About Me (Section 1 of the Toolkit); which included My Story, What Helps Me?, My Goals as a Care Partner, What is My Back-up Plan?, Everyday Hope, and What am I Doing for Myself Today?*,* encouraged them to reflect on their experience as a caregiver:

...you can type down something, type it in, and then it’s almost like a diary. And then kind of go back and go, “Hmm, I wonder, why did I put it that way?

Participant 322, M1

Participants noted that taking time to reflect on their experience was important in dealing with caregiving demands but seldom done:

It [MT4C] allowed me to write down stuff that I haven’t stopped to write down, and I found that that was very helpful...just the opportunity to write down my story and how things have gone. It’s not something a caregiver takes time to do, and it’s really important...It makes you think of stuff that you sort of put in the back of your brain and it makes you put it down in front of you.

Participant 1, M1

The act of reflecting on their experience gave participants an opportunity to analyze their caregiving roles and tasks and assess what changes they needed to make in their thinking or actions related to caregiving:

...it made me take a deeper, inner look at myself, which I seldom do because I’m more focusing on [name of spouse] than I am on myself, and I’ve always found it a little difficult to focus on myself anyhow...it gave me a little chance of soul-searching and analyzing what I am doing, and assessing some of the things I need to revise in my own thinking. So I found it very challenging and interesting.

Participant 301, M1

#### Using My Tools 4 Care Encouraged Sharing of Caregiving Experiences

Participants described how MT4C provided the opportunity to write down their thoughts and share their experiences as a caregiver. For example, caregivers appreciated telling their story, explaining their goals as a care partner, or considering their backup plan. Some caregivers described how MT4C helped them cope with their emotions by allowing them to write about their experiences:

I just found it more, therapeutic, I think, than anything else, to write down those things that I needed to think about.

Participant 383, M1

This was particularly true during stressful situations when other support was not present, as described by one caregiver:

Well, the writing down of the stressful things that were happening; just the fact that I was able to share things and not keep it to myself, kind of thing. As I say, when all this happens, I’m on my own, and just the fact that I can share it is, you know, even if nobody reads it, the fact that I’ve took it out of my mind, there. So I did really find that helpful.

Participant 330, M3

Other caregivers described how MT4C prompted them to be mindful of things that help them get through the day and reinforced the importance of self-care. As the following caregivers stated:

I did do some of the ones [sections of the website] of taking care of myself and...that’s one of my big things is that I understand that I really have to take care of myself, because I can’t help [name of spouse] if I’m not well.

Participant 337, M1

...the place where you had to make a list of the things that help you get through the day, [What Helps Me?] because I think it is such a negative situation and it’s so exhausting, physically and mentally and emotionally, that you could forget about that. So in the sense that it made you sit and think about it, I think that was a positive thing.

Participant 349, M3

**Table 1:** Caregivers’ baseline demographic characteristics (Alberta and Ontario, N=56).

Demographic characteristics		n (%)
**Gender**		
	Male	13 (23)
	Female	43 (77)
**Marital status**		
	Married	47 (84)
	Single	5 (9)
	Divorced/separated	3 (5)
	Common Law	1 (2)
**Ethnicity**		
	White	50 (89)
	Other	6 (11)
**Nationality**		
	Canadian citizen	54 (96)
	Landed immigrant	2 (4)
**Employed**		
	Yes	20 (36)
	No	36 (64)
**Employment status (if employed outside the home)**		
	Full-time	9 (16)
	Casual/part-time	4 (7)
	Not applicable	43 (77)
**Self-employed**		
	Yes	7 (13)
	No	13 (23)
	Not applicable	36 (64)
**Relationship to care receiver**		
	Spouse/partner	31 (55)
	Son/daughter	22 (39)
	Daughter-in-law	2 (4)
	Grand-daughter	1 (2)
**Number of years caring for care recipient^a^**		
	<1 year	14 (25)
	1-2 years	14 (25)
	3-5 years	15 (27)
	6-10 years	10 (18)
	>10 years	3 (5)
**Living with care recipient**		
	Yes	43 (77)
	No	13 (23)
**If yes, type of residence (n=43)**		
	Own home/condo	35 (81)
	Rent home/apartment	7 (16)
	Retirement or assisted living	1 (2)
**Receive assistance with caregiving**		
	Yes	39 (70)
	No	17 (30)
**Caregiver has medical conditions**		
	Yes	44 (79)
	No	12 (21)
**Estimated annual household income before taxes ($ CAD)**		
	$10,000-$29,999	8 (14)
	$30,000-$49,999	14 (25)
	$50,000-$69,999	9 (16)
	Greater than $70,000	19 (34)
	No answer	6 (11)
**Finances meet needs**		
	Completely	7 (13)
	Very well	12 (21)
	Adequately	26 (46)
	With some difficulty	8 (14)
	Not very well/totally inadequately	3 (5)

^a^As of June 1, 2015.

For some caregivers, using MT4C encouraged them to:

...look at what’s coming and plan for the future.

Participant 372, M3

Often, this meant anticipating and planning for the upcoming care needs of the care recipient:

...But the long-term is what made me think...my husband and myself manage all her medical things, and it [MT4C] made me even realize somebody else needs a list of doctors and [chuckles] you know, things like that...It made me think about personal care in the future because that’s long-term care.

Participant 344, M1

Participants also talked about anticipating and planning for changes such as the care recipient’s move to assisted living or long-term care; arranging power of attorney; and anticipating changes to their living arrangements, such as making modifications to their existing home to accommodate the needs of the care recipient, or moving to be closer to family and other forms of support.

#### Using My Tools 4 Care Provided a Source of Information and Education

Caregivers described how MT4C provided them with information or direction on how to find information, for example, about changes to the caregiver’s roles and relationships, environment, physical and mental health, daily activities, and the need for support (Section 2, Common Changes to Expect); how to access services and find information (Frequently Asked Questions, Section 3); and other helpful resources (Section 4, Resources). One participant described MT4C as a reference:

...something I could look at and use part of it or some of it, a little of it or none of it, but it gave me that basis to...sort of a mode of attack of how I was going to handle the situation.

Participant 301, M3

Participants who used MT4C appreciated that it provided useful, timely and new information that helped them to understand and deal with the disease-related changes in the care recipient:

...And even though I did do a lot of research, some of the stuff in there I hadn’t found before, so it helped me.

Participant 11, M1

The information about dementia helped caregivers assess the current stage of dementia and plan for what was to come:

And it [MT4C] gives you the information and very detailed description of each level of, where they’re at in their dementia process. I found that was much better than what other sites that I’ve read...so I was better able to reassess where I thought my husband was at, compared to other sites where I’ve used essentially the same sort of tool, but not worded in such a way that was really as helpful as it is on your site.

Participant 1, M1

Participants found the links to resources within MT4C particularly helpful in supplying specific information that was relevant to planning for taking on new roles:

I like the fact that you give out the telephone numbers and the contact information, national contacts, I think that’s great! That's information worth something to me.

Participant 365, M3

Participants explained that the links to resources, for example information about Power of Attorney, helped them to plan for taking on new roles as caregivers, such as managing financial and health care decisions:

The Resources, that one meant more to me than anything else, that’s probably where I spent most of the time, the links to the legal stuff, I needed that because I wanted to find out about Powers of Attorney.

Participant 350, M3

#### Using My Tools 4 Care Provided Affirmation

Participants described how their use of MT4C provided affirmation that their experiences were comparable to those of other caregivers, that they were not alone in their journeys. Using MT4C provided confirmation that they were doing the best they could in their caregiving roles. This affirmation helped give them the courage to deal with ongoing changes such as taking on new roles and responsibilities as the health of their loved one deteriorated, dealing with increased social isolation, and planning for possible placement. Participants found the examples of transitions experienced by other caregivers available on MT4C (in text or video format) provided:

...confirmation that you're not alone.

Participant 345, M1

Participants repeatedly stated that these written experiences decreased their feelings of isolation and that it was reassuring to know that other caregivers were going through the same situations and feelings.

It’s nice seeing comments from other people, and there should be a lot more of that. Because you end up thinking—and I know it’s not the case—but you end up thinking that you’re the only one going through it, and then you realize that there’s an awful lot of other people doing it, too.

Participant 21, M3

Because dementia caregiving is different for every caregiver and every person with dementia, it was often difficult for caregivers to know if they were using the right caregiving strategies and approaches to care, or if they were making the best decisions for the person with dementia. Caregivers verbalized that MT4C provided a sense of validation that they were providing good care:

Well, basically, [the website validated] that I’m doing the right things, and that I’m providing good care. I’m providing for her comforts and making her quality of life as good as it can possibly be under the circumstances. And that’s [been] my objectives. You know, longevity versus quality [of life].

Participant 23, M3

Caregivers noted that using MT4C reaffirmed the normality of the caregiving journey. Participants felt reassured that their experiences were “normal”:

It’s what’s to be expected, and not anything we didn’t do, or that we’re doing wrong, or, that we haven’t done.

Participant 390, M1

One caregiver used MT4C to validate their caregiving approaches and journey:

In each of the sections, I’ve well been there. I’ve been caregiving since 2008, and I have a large care manual that I started writing, and I’ve accumulated a lot of information...So you know, I have my objectives and I have long-term plans of certain things I’m going to do. So when I look at the website, I think I’m pretty well on track.

Participant 23, M1

#### Using My Tools 4 Care Did Not Help

Some participants indicated that using MT4C did not help them because (1) they were meeting or had already met their caregiving needs by other means, (2) they felt that it was not the right time to use MT4C, or (3) they did not have time to use MT4C. Many participants in this study were experienced caregivers; 50% had been caregiving for 3 or more years. Therefore, many had long established strategies in place to help meet their caregiving needs. These were often cited as reasons for not using parts of MT4C. For example, several participants indicated that they were already familiar with available resources and had already used or were using community-based services to answer their questions and address their caregiving concerns. As one participant explained:

It [MT4C] didn’t help me significantly...I had gone to some caregivers’ group and got some information there.

Participant 24, M3

Many caregivers had contacted a local branch of the Alzheimer Society in the past or were currently connected with the organization and attending various education or support groups or obtaining more immediate advice by phone:

I’ve been extremely reliant on the Alzheimer Society...and I’ve found them to be very good with their information and their support...I think most of the time...we have something already in place.

Participant 308, M3

In some cases, participants felt that they didn’t really need MT4C or that the information provided in MT4C did not apply to their current situation, indicating that it was either too early or too late in their dementia journey to use MT4C. This is evident from the following statements:

...didn’t really need it [MT4C] at this point.

Participant 379, M3

I feel like I’m not there yet; Mom’s still early, so some of the things are a bit more advanced, talking about getting help and that sort of thing. We aren’t at that stage yet so I could see maybe as things progress that maybe I’d be going back here to kind of have it as another resource. I think that’s probably the main thing, is I feel like I don’t need it yet.

Participant 345, M1

One caregiver who had been caring for her 89-year-old mother for almost 4 years expressed how she felt that the Toolkit could not help her because:

I’ve figured out everything on my own.

Participant 35, M1

The same participant stated:

...I’m at the end now. And for somebody new into the dementia journey, I think it’s a great tool...right now, because I’m emotionally wrecked, physically, financially it [MT4C] can’t help me now [laughs].

Participant 35, M3

#### Features of My Tools 4 Care Found to Be Most and Least Helpful

Caregivers found the layout of MT4C to be “very well organized” [Participant 342, M3], easy to navigate, and easy to use. They also found it was helpful to (1) have the opportunity to reflect on and to share their caregiving experiences (in writing); (2) receive information that was relevant and applicable to their situation; and (3) obtain validation of their caregiving experiences through the content of the website and linked videos. Aspects of the Toolkit that some participants found were least beneficial included (1) the Toolkit did not apply to the caregiver’s current situation or suit their current needs because of their stage in the caregiving journey; (2) challenges with technology and security concerns; (3) writing or sharing their thoughts and experiences in MT4C; and (4) lack of time to use the site due to the demands of caregiving and other responsibilities. As one caregiver explained:

The amount of time you had to sit and write things down, type things in, and to be honest with you, the more time I spend on the computer, the more [name of spouse] approaches me and saying “What are you doing? Why aren’t you sitting with me?”

Participant 353, M3

#### Caregivers’ Recommendations to Improve My Tools 4 Care

Participants offered a number of recommendations to improve the content and format of the Toolkit and other Web-based resources for caregivers. Suggestions for guidance and information about local resources and how to access them were commonly expressed by participants. To meet these needs, caregivers suggested (1) adding a directory of services that is searchable by postal code, (2) having a person available to answer caregivers’ questions by telephone, and (3) having a navigator to “be that bridge” [Participant 373, M3] to help the caregiver identify and access resources that meet their specific needs.

Participants also requested practical caregiving tips and strategies to help them manage the daily challenges they face as caregivers of older adults with ADRD and MCC and particularly valued receiving this information from other caregivers. Some participants commented that adding a feature to MT4C to enable caregivers to connect with one another to share information, experiences, and caregiving strategies would be helpful:

...if you connect with people over the internet say, you know, I’m having a really hard time today and somebody can say: “I know what you’re going through,” that can be good support too, you know?

Participant 399, M3

Some caregivers suggested improvements to make MT4C more user-friendly. These included reducing the use of medical language and adjusting literacy levels and providing an overview of the content of the site. As one participant stated:

...it is a lot of text, and the literacy level. Oh, the other thing is it’s only in English...you need to make the language a bit simpler.

Participant 331, M3

## Discussion

### Principal Findings

This is the first known study to examine how a Web-based intervention helps caregivers of community-dwelling older adults with ADRD and MCC. Caregivers indicated that using MT4C encouraged reflection, helped them to share their caregiving experiences, provided information and resources and affirmed their caregiving roles. This, in turn, helped them to deal with caregiving demands such as taking on more roles and responsibilities as their loved one’s health and abilities declined; coping with increasing social isolation; caring for self as their own health declined, and planning for future caregiving changes such as arranging alternate living arrangements.

MT4C served as a confidential outlet that provided participants a rare opportunity to tell their stories and to reflect on their caregiving experiences through writing. Caregivers were prompted to think about aspects of caregiving that they had not considered before, including their own health and well-being and the importance of self-care. In some cases, the act of reflecting on and writing about their experiences resulted in new insight that helped to reduce caregivers’ stress and helped them to cope with the challenges and emotions associated with their role. Other authors have reported positive psychological benefits of writing. For example, family caregivers of people with dementia who were randomly assigned to a poetry-writing intervention experienced a sense of accomplishment in both writing a poem and caring for their family member [32]. They also reported a sense of catharsis, or emotional release, increased acceptance of their (caregiving) situation or for their care recipient, and greater self-awareness [32].

Participants valued MT4C as an accessible and reliable source of meaningful information and resources that could help them with their caregiving roles. They described the benefit of knowing more about dementia and the changes to expect in their loved one and their own roles and responsibilities. They also emphasized the value of links to information about topics such as legal issues and planning for placement. These findings are consistent with other research related to Web-based supports where caregivers expressed the importance of meaningful resources to support their roles [19].

The links to videos and quotes describing other caregivers’ experiences provided affirmation to many MT4C users and, thereby, reduced feelings of isolation and provided a sense of connection with other caregivers. Other studies of Web-based interventions for caregivers confirm the importance of connections with other caregivers [20,22].

Participants also identified aspects of the Toolkit that were not beneficial. Not all caregivers liked to focus on themselves, to write out their personal thoughts, or to share their caregiving experiences in MT4C. Some caregivers chose to learn and share their experiences by other means, usually by attending a support group for caregivers, or speaking one-to-one with a counselor. Previous research related to Web-based supports indicates that caregivers value the opportunity to connect with professionals to support them in their caregiving [18,22].

Some caregivers felt that MT4C did not apply to their current situation or suit their needs, noting that it was either too early or too late in their caregiving journey to benefit from using MT4C. Some caregivers felt they had already dealt with an issue that was addressed by MT4C or had already obtained information from other sources, most often a local branch of the Alzheimer Society. In the pilot study of the Toolkit [25], participants suggested that it would be of most use to those in the early stages of caregiving; however, the findings of this study suggest that, in addition to the timing of the intervention, a caregiver’s perceived need for support and information via a psychosocial Web-based intervention is important.

Participants were challenged with balancing multiple responsibilities and demands on their time, including, and in addition to, the care of an older adult with ADRD and MCC, yet they valued and learned from other caregivers’ experiences (eg, videos and written comments) and suggested that future Web-based tools should include a means of connecting with other caregivers to share knowledge and experiences. These findings are congruent with recent qualitative evidence that demonstrates that Web-based social interventions benefit caregivers of persons with dementia by promoting sharing and companionship and reducing social isolation [13].

### Implications

There are a number of implications for future practice and research related to Web-based interventions for caregivers of persons with ADRD and MCC arising from this study. Study results suggest that use of Web-based resources, such as MT4C, offer valuable support to help caregivers as they experience a range of complex caregiving roles and responsibilities. Findings indicate that future Web-based resources for caregivers should contain (1) opportunities for reflection on their caregiving journey and self-care; (2) options to share their caregiving experiences; (3) links to resources (eg, community health and support services, legal information) that they could use in planning for future caring; and (4) links to stories and videos of other caregiver experiences.

Participants appreciated that MT4C provided the opportunity to reflect on and write about their experiences as a caregiver and to learn from other caregivers through linked videos. Some participants suggested that adding a feature to MT4C to enable caregivers to connect with one another (in real time or asynchronously) to share information, experiences, and caregiving strategies would be helpful. Incorporation of such features should be considered in the future development of Web-based supports for caregivers.

This Web-based intervention was developed with the theoretical lens of transitions theory [26] and thus, provides greater insight into the multiple, concurrent transitions experienced by caregivers who are caring for an older adult with ADRD and MCC. The application of transitions theory also facilitates the ongoing refinement of strategies to best meet caregivers’ personalized needs. Future development of Web-based resources should consider a theory-based approach and how to best meet the complex transition-related needs of caregivers of individuals with both ADRD and MCC [33].

Many participants in this study had previously accessed or were currently using various community-based resources for caregivers of persons with ADRD. The resources listed in MT4C were focused on national or provincial services, and future development of Web-based resources could consider adding more locally available resources, perhaps with geospatial mapping.

Future research on Web-based supports such as MT4C could examine the perceptions of caregivers with a broader cultural and language diversity. Research is also needed to better understand the impact of Web-based supports for caregivers of persons with dementia and MCC when used in combination with other forms of support, including professional and peer support or telephone support.

### Limitations

Currently, MT4C is only available in the English language and therefore, the sample showed little ethnic or cultural diversity. Follow-up beyond 3 months would have been useful to understand caregivers’ use of the Web-based tool over a longer period of time.

### Conclusions

Study results indicate that a self-administered psychosocial supportive Web-based resource helps caregivers of community-dwelling older adults with ADRD and MCC deal with their caregiving responsibilities. In particular, caregivers indicated that use of MT4C encouraged reflection, helped them to share their caregiving experiences, provided information and resources, and affirmed their caregiving roles. This, in turn, helped them to deal with caregiving roles and responsibilities, identifying supports for caring, caring for self, and planning for future caregiving roles and responsibilities. There is a need for further research in the field of Web-based supports for caregivers of older persons with ADRD and MCC as they have great potential as accessible and cost-effective ways to improve the well-being of these caregivers.

## Abbreviations

ADRD: Alzheimer disease and related dementias
MCC: multiple chronic conditions
MT4C: My Tools 4 Care or Transition Toolkit or Toolkit
RCT: randomized controlled trial
